# Effect of *L. plantarum *cell-free extract and co-trimoxazole against *Salmonella *Typhimurium: a possible adjunct therapy

**DOI:** 10.1186/1476-0711-10-9

**Published:** 2011-02-27

**Authors:** Praveen Rishi, Simran Preet, Prabhjot Kaur

**Affiliations:** 1 Department of Microbiology, Basic Medical Sciences Block, Panjab University, Sector-14, Chandigarh-160014, India

## Abstract

**Background:**

Frequent and indiscriminate use of antibiotics has led to the development of multi-drug resistant bacterial strains. It necessitates the exploitation of alternative therapeutic strategies. In order to reduce the dose of antibiotic required and to decrease the associated side effects, the present study was aimed at evaluating the synergism, if any, between a conventional antibiotic, co-trimoxazole (CTZ)) and cell free supernatant (CFS) of a probiotic (*L. plantarum) *against *S. *Typhimurium NCTC 74. This antimicrobial combination was selected on the basis of antibiotic susceptibility pattern of *Salmonella *and *L. plantarum*.

**Methods:**

The synergy was evaluated in terms of size of zone of inhibition, fractional inhibitory concentration index, time-kill assay (*in-vitro) *as well as macrophage functions (*ex-vivo*).

**Results:**

The concentration producing the same or higher antibacterial effect (size of zone of inhibition) was reduced to half when both the agents were used in combination with respect to the concentrations required when used separately. CTZ and CFS exhibited synergetic activity against *Salmonella *by checkerboard microtitre test and the time-kill test. *Ex-vivo *studies demonstrated a significantly higher intracellular killing of bacteria by macrophages treated with CFS (80 AU/ml) + (CTZ) (2 μg/ml) as compared to when treated with both separately at higher concentrations. Significant reduction in the extent of lipid peroxidation and nitrite levels generated by macrophages in presence of CFS and CTZ, in conjunction, further substantiated the synergistic efficacy of the combination.

**Conclusions:**

The antimicrobial efficacy of this combination indicates that it may serve as the basis in developing alternative strategies to combat *Salmonella *infections.

## Background

*Salmonella *infections cause a great deal of morbidity and mortality worldwide, especially in developing countries because of improper sanitary conditions and inadequate health facilities. Antibiotics have been the mainstay of therapy to combat *Salmonella *infections. However, the use of antibiotics is under question due to complications involving the emergence of multidrug (MDR) resistant strains of *Salmonella *[1,2]. Besides this, frequent and lengthy use of antibiotics usually results in alteration of the intestinal commensal flora [3] and lead to chronic toxicity. It necessitates the exploitation of alternative antibacterial therapies against *Salmonella *infections. Synergistic combinations of antibiotics and other antimicrobials may be effective not only against infections where the development of resistance and/or subsequent failure to monotherapy is prevalent but also in prevention of emergence of bacterial resistance [4]. One such alternative is the possible therapeutic use of probiotics as an adjunct to chemotherapy [5].

Probiotics are dietary supplements containing potentially beneficial bacteria or yeasts. According to the currently adopted definition by FAO/WHO in 2001, probiotics are 'live microorganisms, which when administered in adequate amounts confer a health benefit on the host' [6]. The role of probiotics has been reported in prevention and treatment of gastrointestinal infections caused by *Salmonella *[7,8], rotavirus and *Clostridium difficile *[9-11]. Probiotics in combination with antibiotic treatment have been reported to be successful in the management of *Helicobacter pylori *infection [12].

Probiotic and antibiotic combinatorial therapy may provide higher antimicrobial activity and reduce the dose of antibiotic required besides replenishing the intestinal flora thereby providing benefit to the host [13]. The present study was therefore carried out to assess the in-vitro and ex-vivo synergistic effect, if any, of cell-free *Lactobacillus plantarum *supernatant (CFS) in conjunction with co-trimoxazole (CTZ) against *Salmonella *Typhimurium.

## Methods

### Bacterial strains and growth conditions

*Lactobacillus casei *MTCC 1423, *Lactobacillus plantarum *MTCC 2621 and *Lactobacillus acidophilus *MTCC 447, standard LAB strains were procured from Institute of Microbial Technology (IMTECH), Chandigarh, India. *Salmonella enterica *serovar Typhimurium NCTC74 was procured from the Central Research Institute (CRI), Kasauli. This strain has been used in previous studies both as a virulent strain [14] and as a reference strain [15].

### Growth Conditions

*Salmonella *Typhimurium was maintained on Brain Heart Infusion Broth and sub-cultured bimonthly. It was grown on nutrient broth for 18 h at 37°C. After centrifugation, the culture was harvested in phosphate-buffered saline (PBS) and adjusted to a final cell count of approx. 4 × 10^7^CFU/ml. Lactobacilli strains were grown in De Mann Rogosa Sharpe (MRS) broth for 24 h and adjusted to a final concentration of approx. 9 × 10^8^CFU/ml. The bacterial strains used in the present study were confirmed by their morphological and biochemical characteristics.

### Antibiotic susceptibility tests

All procured strains were tested for their susceptibility to various antibiotics (μg): Cotrimoxazole (25); Gentamycin (10); Ampicillin (25); Cephalexin (30); Norfloxacin (10); Amikacin (30); Cefoxitin (10); Chloramphenicol (10); Cefuroxine (30); Augmentin (10); Cefotaxime (30); Ciprofloxacin (10) and Tetracycline (25) by the method of Bauer et al. [16]. 10^6 ^colony forming units (CFUs) of each Lactobacillus strain were spread plated individually onto MRS agar plate. Hi-media octadiscs impregnated with antibiotics were placed on the surface of the agar plates. The plates were incubated at 37°C for 18-24 hrs and examined for the zones of inhibition appearing around each antibiotic disc. The experiment was repeated thrice and the average inhibitory zone diameters were compared with the standards provided by the National Committee for Clinical Laboratory Standards (NCCLS) [17]. Antibiotic sensitivity of *Salmonella *Typhimurium was also determined by the same method using nutrient agar plates.

### Agar well diffusion assay (agar-WDA)

As the antibiotic susceptibility pattern indicated that *Lactobacillus plantarum *was resistant and *Salmonella *Typhimurium was sensitive to co-trimoxazole, the feasibility of using this combination was further confirmed by agar-WDA [18]. 10^7 ^CFU of *L. plantarum *and *S. *Typhimurium were spread plated on MRS and nutrient agar plates respectively. Wells of 5 mm diameter were punched in the agar plates and filled with 20 μl co-trimoxazole stock solution (1 mg/ml). The plates were then incubated at 37°C for 24 h and observed for the zones of inhibition around the wells.

### Preparation of cell free supernatant (CFS) from *L. plantarum*

CFS was prepared as described by Ogunbanwo et al. [19]. *L. plantarum *was propagated in 1L MRS broth (pH 6.5) for 24 hours at 37°C. Cell free supernatant obtained by centrifuging the culture at 10,000 rpm for 20 minutes at 4°C was filtered through 0.22 μm pore size cellulose acetate filter.

### Quantification of antagonistic activity of CFS against *Salmonella *Typhimurium

The antibacterial activity of CFS against *S. *Typhimurium was tested at twofold serial dilutions using agar-WDA as described above. For this experiment, wells were filled with 10 μl of each CFS dilution and the antibacterial activity was expressed in terms of arbitrary unit (AU). It was defined as the maximum dilution which produced a minimum of zone that still gave a clearly visible antibacterial zone. The reciprocal of the dilution gave the titre of antibacterial activity in AU per mm [20].

### *In-vitro *inhibitory effect of CFS and co-trimoxazole

#### Agar-well diffusion assay

Nutrient agar plates were spread plated with approx. 10^6 ^CFUs of *S. *Typhimurium and the wells were filled with 20 μl solution containing various concentrations of CTZ (10 μg/ml, 20 μg/ml, 30 μg/ml) and CFS (12 AU/ml, 16 AU/ml and 20 AU/ml) separately as well as in combination of both at half the concentrations i.e. (CTZ (5 μg/ml) + CFS (6 AU/ml); CTZ (10 μg/ml) + CFS (8 AU/ml) and CTZ(15 μg/ml) + CFS (10 AU/ml). After incubation at 37°C for 24 h, diameter of the clear zones around the wells was measured.

#### Determination of minimum inhibitory concentrations (MICs)

The minimum inhibitory concentrations (MICs) of CFS and co-trimoxazole against *S. typhimurium *were determined by broth micro-dilution method. Co-trimoxazole powder (Torque Pharmaceuticals, India) was dissolved at a maximum concentration of 1 mg/ml in distilled water for MIC determination. The maximum concentration of CFS was 1280 AU/ml for MIC determination. Co-trimoxazole and CFS were subjected to a two-fold dilution series with Mueller-Hinton Broth and LB medium respectively. 10 μl of each diluted solution was added in micro-titre plate wells. Then 90 μl of *S. *Typhimurium cell suspension adjusted to 10^6^cfu/ml was added to the wells and microtitre plate was incubated for 24 h at 37°C. The lowest concentration that completely inhibited microbial growth as determined by optical density measurements at 600 nm was taken to be the MIC.

#### Synergistic activity of co-trimoxazole and CFS on checkerboard micro-titre test

Checkerboard test was performed in 96-well micro-titre trays using an 8·8 well configuration. Eight 2-fold serial dilutions of co-trimoxazole and CFS were prepared with concentrations ranging from 0 to 2 MIC. 10 μl of CTZ dilution was added to the wells of a 96-well plate in a vertical orientation and 10 μl of each CFS dilution was added in a horizontal orientation so that the plate contained various concentrations of combinations of the two compounds. Then each well was supplemented with 80 μl (10^6 ^CFU/ml) of *S. *Typhimurium and the plate was incubated at 37°C. Wells not containing any antibacterial agent were used as the positive growth controls. Fractional inhibitory concentration (FIC) was calculated by dividing the MIC of the combination of CFS and CTZ by the MIC of CFS or co-trimoxazole alone. The FIC index (FICI), obtained by adding both FIC, was interpreted as indicating a synergistic effect when it was ≤ 0.5, as additive or indifferent when it was > 0.5 and ≤ 2.0, and as antagonistic when it was > 2.0 [21].

#### Time-kill Assay

To determine the bactericidal action of co-trimoxazole and CFS, separately and in combination, *S. *Typhimurium was exposed to one of the antimicrobial agents or to both simultaneously and the viable count was monitored. Co-trimoxazole (2 μg/ml), CFS (80 AU/ml) and their combination were added to different 20 ml nutrient broth flasks containing 10^6 ^CFUs of *S. *Typhimurium and incubated at 37°C. 100 μl aliquots were withdrawn at at 0 h, 4 h, 8 h, 12 h, 24 h, 28 h, 32 h, 36 h, 48 h and spread plated on MacConkey agar plates. The plates were incubated at 37°C for 24 hours for enumeration of colony forming units.

### *Ex-vivo *effect of CFS and co-trimoxazole

Extraction of peritoneal macrophages: Murine peritoneal macrophages were isolated by the method as described by us earlier [14].

### Intracellular killing of S. Typhimurium

Mouse peritoneal macrophages were infected with *S. *Typhimurium at a multiplicity of infection of 1:100. Extensively washed infected macrophages were treated with minimum inhibitory concentration of both i.e. CFS (640 AU/ml) and co-trimoxazole (8 μg/ml) separately and in combination as per fractional inhibitory concentrations i.e. (CFS (80 AU/ml) + CTZ (2 μg/ml)). After every 30, 60 and 90 min of treatment period, treated and untreated macrophages were pelleted (2000 rpm, 10 min) and lysed with 500 μl of 0.25% TritonX-100. Lysates were serially diluted and plated on MacConkey agar medium. After an incubation of 24 h at 37°C, CFUs were counted [14].

#### Interaction of macrophages with *S. *Typhimurium

To assess the extent of lipid peroxidation and to measure the nitric oxide generation, murine peritoneal macrophages were interacted with *Salmonella *(MOI of 1:100) in various groups: **Group A**: 1.5 ml of macrophage suspension (10^5^/ml) + 0.8 ml of *S. *Typhimurium cell suspension (10^7^/ml); **Group B**: 1.5 ml of macrophage suspension (10^5^/ml) + 0.8 ml of *S. *Typhimurium cell suspension (10^7^/ml) + CFS (640 AU/ml); **Group C**: 1.5 ml of macrophage suspension (10^5^/ml) + 0.8 ml of *S. *Typhimurium cell suspension (10^7^/ml) + CTZ (8 μg/ml); **Group D**: 1.5 ml of macrophage suspension (10^5^/ml) + 0.8 ml of *S*. Typhimurium cell suspension (10^7^/ml) + CFS (80 AU/ml) + CTZ (2 μg/ml) for 16 h in a six-well tissue culture plate at 37°C in CO_2 _incubator for 18 hrs. After incubation, lysis buffer (20 mM Tris-HCl, 150 mM NaCl, 1 mM EDTA, 1% Triton X-100, 1 mM PMSF) was added (1:1 ratio) to all the wells and the plate was further incubated at 4°C for 20 min. Reaction mixtures form each well were centrifuged (2,000 rpm, 15 min) and supernatant thus obtained was used to study the following:

### Extent of lipid peroxidation

Quantitative measurement of lipid peroxidation in the culture supernatants of macrophages was performed as described by us earlier [22]. The results were expressed as nanomoles of MDA per milligram of protein, using the molar extinction coefficient of chromophore (1.56×10^5^M^-1 ^cm^-1^). Protein content of the samples was estimated by the method as described by Lowry et al. [23].

### Estimation of nitrite concentration

The amount of nitric oxide in cell-free supernatant was determined by slight modification of the method of Green et al. [24] as described by us earlier [25]. 100 μl aliquots of sample were mixed with 400 μl of distilled water and 500 μl of Griess reagent. The reaction mixture was incubated at room temperature for 10 min. (in dark) and optical density was measured at 546 nm. Nitrite was quantified using standard graph of sodium nitrite.

### Statistical analysis

Results were expressed as mean ± standard deviation (SD). The inter group variation was assessed by one way analysis of variance (ANOVA) followed by Fischer's LSD test. Statistical significance of the results was calculated to at least P < 0.05.

## Results

### Antibiotic sensitivity pattern of Lactobacilli and *S. T*yphimurium

A compatible probiotic-antibiotic combination was found to be that of *L. plantarum *and co-trimoxazole. *S. *Typhimurium was found to be sensitive to co-trimoxazole while amongst the three Lactobacilli tested, *L. plantarum *was found to be resistant to the same (Table 1).

**Table 1:** Antibiotic susceptibility pattern of test Lactobacilli strains i.e *L. plantarum*, *L. casei*, *L. acidophilus *and indicator strain *S*. Typhimurium as determined by disc-diffusion technique

Antibiotics	Conc. ug/disc	Test Lactobacillus strains			Indicator strain *S*. Typhimurium
		***L. acidophilus***	***L. casei***	***L. plantarum***	
Norfloxacin	10	R	R	R	S
Cotrimoxazole	25	S	S	R	S
Gentamycin	10	S	R	S	S
Ampicillin	25	R	S	R	R
Cephalexin	30	S	S	S	S
Amikacin	30	S	R	R	S
Cefoxitin	10	R	R	R	R
Chloramphenicol	30	S	S	R	S
Cefuroxine	30	S	R	R	S
Augmentin	10	R	R	R	R
Tetracycline	25	S	S	S	S

S-sensitive R-resistant

### Agar well diffusion assay (agar-WDA)

**Figure 1:**
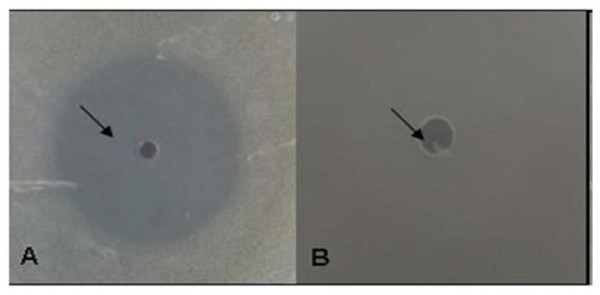
**Agar-well diffusion assay**. **(A) **Zone of inhibition of growth of *S. *Typhimurium produced by co-trimoxazole (10 μg/ml) **(B) **Resistance exhibited by *L. plantarum *to co-trimoxazole (10 μg/ml) (agar-well diffusion assay)

The sensitivity of *S. *Typhimurium and resistance of *L. plantarum *to co-trimoxazole was further confirmed by agar-WDA. A clear zone of inhibition (15 mm) of growth of *S. *Typhimurium (Figure 1A) could be observed while *L. plantarum *exhibited complete resistance to co-trimoxazole (Figure 1B). These observations indicated the feasibility of using CFS of *L. plantarum *in conjunction with co-trimoxazole against *S. *Typhimurium NCTC74.

## Quantification of the antagonistic activity of *L. plantarum *cell-free supernatant (CFS)

A clear zone of growth inhibition could be observed around the wells containing CFS indicating that *L. plantarum *was inhibitory to the growth of *S. *Typhimurium. The maximum dilution of the cell-free supernatant that produced a minimum of antibacterial zone was 1: 1600. It was therefore estimated that the antagonistic activity of the cell-free supernatant was 1600 AU/ml.

## Agar-WDA

**Figure 2:**
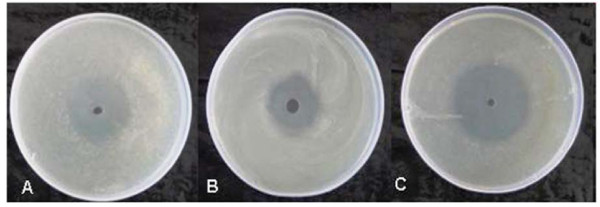
**(A-C)-Synergy exhibited by CTZ and CFS in terms of size of zones of inhibition produced when used separately and in combination against *S. *Typhimurium as determined by agar-WDA**. (A) CTZ (10 μg/ml) (B) CFS (12 AU/ml) (C) CFS (6 AU/ml) + CTZ (5 μg/ml)

When the wells were filled with 12 AU/ml, 16 AU/ml and 20 AU/ml of CFS alone, the zone sizes obtained were 22 mm, 28 mm and 33 mm respectively. Similarly, co-trimoxazole at 10, 20 and 30 μg/ml concentrations produced a zone size of 24, 31 and 40 mm respectively. However, interestingly in combination at half the concentrations of both agents i.e. (CTZ (5 μg/ml) + CFS (6 AU/ml); CTZ (10 μg/ml) + CFS (8 AU/ml); CTZ (15 μg/ml) + CFS (10 AU/ml), the zone sizes were 32, 34 and 44 mm respectively (Figure 2A-C are representative figures). The observed zone of growth-inhibition of *Salmonella *Typhimurium indicated synergy between the two as the dose required to give the same antibacterial effect in terms of size of zone of inhibition was reduced to approximately half when CFS and co-trimoxazole were used in combination as compared to when used individually at different concentrations.

## MICs of co-trimoxazole and CFS

The MICs of cotrimoxazole and CFS against *Salmonella *Typhimurium were evaluated to be 8 μg/ml and 640 AU/ml respectively.

## Fractional inhibitory concentrations (FIC) of co-trimoxazole and CFS

The inhibitory activity of the combination of co-trimoxazole and CFS was determined by the FIC index at 64 different combinations. FICI index was ≤ 0.5 indicating that there is synergistic effect of the combination. When used together, co-trimoxazole and CFS inhibited the growth at four fold (2 μg/ml) and eight fold (80 AU/ml) lower concentrations than when applied alone.

## Time Kill Assay

**Figure 3:**
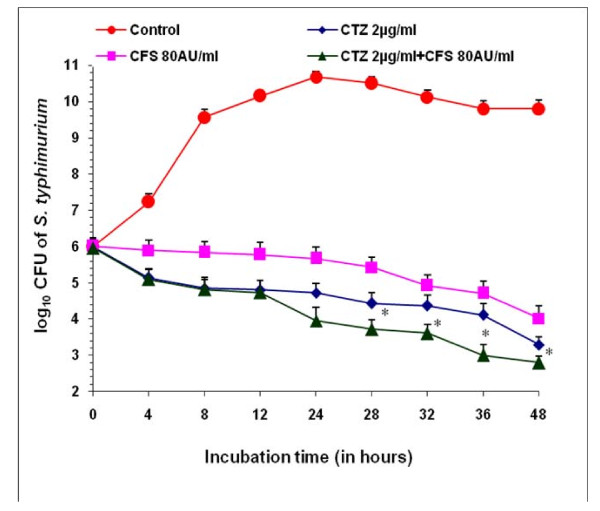
**Time-kill assay**. Log_10 _CFU of *Salmonella *Typhimurium NCTC 74 at various time intervals in presence of cotrimoxazole (2 μg), CFS (80 AU/ml) alone and in combination i.e CTZ(2 μg/ml) + CFS (80 AU/ml). Values are expressed as mean ± S.D. of three individual values. *p < 0.05 vs. CTZ (10 ug/ml) and CFS (12 AU/ml).

After 48 hours, CFS (80 AU/ml) and CTZ (2 μg/ml) separately gave a decrease of 5.78 log units and 6.5 log units respectively as compared to control (48 h). However, when used in combination, (CFS (80 AU/ml) + CTZ (2 μg/ml), a decrease of 7 log units was observed after 48 h (Figure 3) indicating the synergetic effect.

## Effect on intracellular killing of *S. *Typhimurium

The mean percentage intracellular killing in presence of CFS alone at 30, 60 and 90 minutes was 13.7%, 35% and 48.75% respectively, at p < 0.05. Similarly, when infected macrophages were treated with co-trimoxazole alone, the mean intracellular killing was 21.25%, 42.5% and 63.75% at 30, 60 and 90 minutes respectively (p < 0.01). A higher intracellular killing was observed when infected macrophages were treated with co-trimoxazole in conjunction with CFS. In this case, the killing was found to be 25% (†p < 0.01), 53.75% (†p < 0.01) and 92.3% (‡p < 0.001) at 30', 60' and 90' respectively (Table 2). On the other hand, the intracellular killing in untreated macrophages was 11.2%, 27.5% and 43.75% at 30, 60 and 90 minutes respectively. The results indicate that both CFS and co-trimoxazole might act cooperatively leading to an enhanced killing of intracellular *S. *Typhimurium.

**Table 2:** Effect of CTZ and CFS on intracellular killing of *Salmonella *Typhimurium NCTC 74 (assessed by isolating murine peritoneal macrophages from Balb/c mice and interacting them with *Salmonella *in presence of CFS and CTZ separately and in combination)

Duration of intra-cellular incubation (min.)	CFU of *S. *Typhimurium				Mean Percentage Intracellular Killing {(No-N_t_/N_0_)×100}			
	
	Untreated Macrophages	Treated Macrophages			Untreated Macrophages	Treated Macrophages		
		CTZ	CFS	CTZ + CFS		CTZ	CFS	CTZ + CFS
0 (N_0_)	80×10^7^	-	-	-		-	-	-
30	71×10^7^	63×10^7 *^	69×10^7^	60×10^7 *†^	11.2	21.25	13.7	25
60	58×10^7^	46×10^7 *^	52×10^7 *^	37×10^7 *†^	27.5	42.5	35	53.75
90	45×10^7^	29×10^7 *^	41×10^7 *^	6.1×10^7 *‡^	43.75	63.75	48.75	92.3

Co-trimoxazole-CTZ; Cell free supernatant from *L. plantarum *- CFS.

## Estimation of lipid peroxidation (MDA) levels

**Figure 4:**
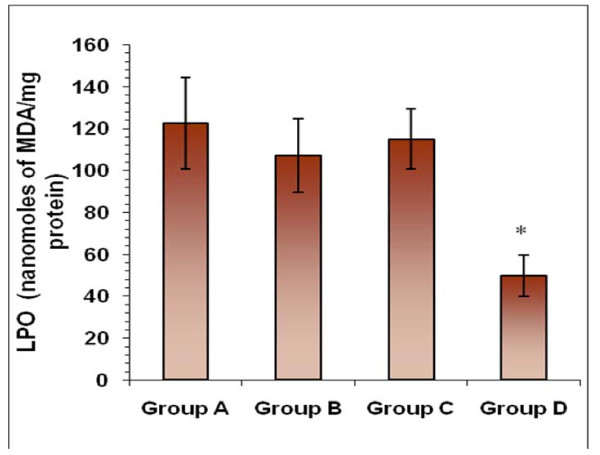
**Estimation of lipid peroxidation (MDA) levels**. Effect on the levels of lipid-peroxidation (in terms of MDA levels) of macrophages when infected with *S. *Typhimurium in presence of co-trimoxazole and CFS alone and in combination. Values are expressed as mean ± S.D. of four individual values. *p < 0.05 vs. Group A

No significant decrease in the levels of MDA was observed when macrophages were interacted with *S. *Typhimurium *i*n the presence of CFS and co-trimoxazole alone as compared to the normally interacted macrophages (in absence of any antimicrobial agent). However, MDA levels were significantly reduced where macrophages were interacted with *S. *Typhimurium in the presence of CFS and co-trimoxazole in combination (Figure 4).

## Estimation of nitrite levels

**Figure 5:**
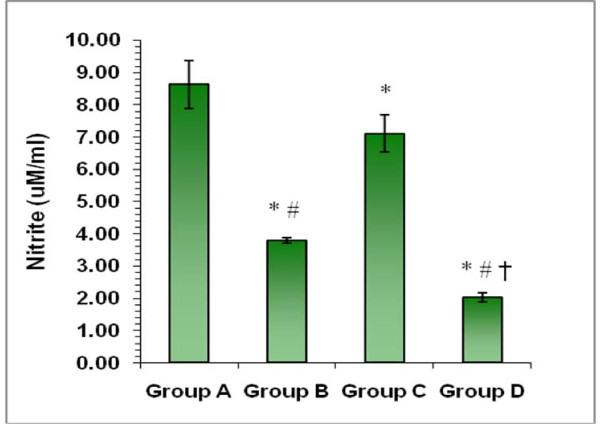
**Estimation of nitrite levels**. Effect on the levels of nitrite levels of macrophages when infected with *S. *Typhimurium in the presence of co-trimoxazole, CFS alone or in combination. *p < 0.05 vs. Group A; **^≠^**p < 0.05 vs. Group C; ^†^p < 0.05 vs. Group B.

Significant decrease (p < 0.01) in the levels of nitrite reduced by macrophages was observed in presence of cotrimoxazole as compared to the normal infected macrophages. Similarly, nitrite levels of infected macrophages were significantly reduced in the presence of CFS also but to a lesser extent (p < 0.05) (Figure 5). However, a more pronounced effect (p < 0.01) was observed when macrophages were interacted with *S. *Typhimurium in the presence of both CFS and co-trimoxazole together.

## Discussion

Therapy with antimicrobial combinations has been in use for a long time and is often applied to take advantage of different mechanisms of action of the agents involved [13]. Co-trimoxazole, a combination of a sulfamethoxazole with trimethoprim, is a synergistic combination, which has been used to treat Salmonellosis [26]. However, some *Salmonella *strains have been reported to develop resistance to co-trimoxazole [27,28]. Consequently, the study of co-trimoxazole in combination with probiotics might prove useful as frequency of development of microbial resistance to the combination is likely to be lower than it would be to either agent, if used alone. Therefore, the current study was carried out to detect whether cell free supernatant from *L. plantarum *in combination with co-trimoxazole therapy would have higher antimicrobial activity against *S. *Typhimurium.

Keeping in view the application of probiotics to be used in conjunction with an antibiotic, the first criteria which a probiotic strain needs to fulfil is that it should be resistant to that particular antibiotic to avoid the direct killing of the probiotic strain. After evaluating the resistance and sensitivity of *L. plantarum *and *S. *Typhimurium respectively to co-trimoxazole, the combination of cell free supernatant from *L. plantarum *and co-trimoxazole was further tested to evaluate the synergistic effect against *S. *Typhimurium. The results exhibited synergy between the two agents as the dose required to give the same antibacterial effect in terms of size of zone of inhibition and colony forming units was reduced to approximately half as compared to when used individually at higher doses. CFS contains several antimicrobials [29], lactic and non-lactic acids as well as hydrogen peroxide which kill pathogens [30] while co-trimoxazole is known to interfere with bacterial folate synthesis [31]. These two mechanisms might have acted cooperatively with each other leading to a higher bactericidal effect of the combination. FIC index also further substantiated the synergism between the two.

The *in-vitro *synergism observed between CFS and co-trimoxazole prompted us to investigate the ex-vivo synergistic efficacy in terms of macrophage functions keeping in view the intracellular survival ability of *Salmonella*. It is also known that *S. *Typhimurium infection of macrophages increases intracellular reactive oxygen intermediates (ROIs), which in turn increase the expression of other antimicrobial factors [32]. Thus, significantly enhanced killing of the *Salmonella *in the presence of CFS and co-trimoxazole, in combination, might be due to the cooperativity between the macrophage antibacterial effectors and the antimicrobial combinations used [14]. Corroborating with the present data, earlier studies have also shown that cell free supernatants and/or extracts from lactic acid producing bacteria may activate macrophages *in-vitro *thereby resulting in augmented phagocytic activity [33,34].

Further, a significant decrease in extent of lipid peroxidation (in terms of MDA level) was also observed in the presence of CFS and co-trimoxazole both. This indicated that the combination of cell free supernatant and co-trimoxazole may exhibit the synergistic effect by scavenging free radicals thereby providing protection against oxidative damage [7]. In support of our findings, Lin and Yen [35] have also reported the inhibition of lipid peroxidation by cell free extracts of *L. acidophilus *and *B. longum *using lipid model systems.

Nitric oxide is an important signalling molecule which acts in many tissues to regulate diverse range of physiological process. The estimation of nitrite is an indirect measure of nitric oxide content. In the present study also, a significant decrease in nitrite levels was observed when macrophages were interacted with *S. *Typhimurium in presence of both co-trimoxazole and CFS as compared to infected macrophages in the absence of the agents. It may be attributed to the enhanced ability of probiotics to attenuate TNF-α or TNF-α stimulated IL-8 production in presence of co-trimoxazole, which might result in further decrease of NO levels [30,36].

## Conclusions

In conclusion, the *in-vitro *and *ex-vivo *studies clearly indicate that cell-free extract from *L. plantarum *and co-trimoxazole act synergistically against *Salmonella *Typhimurium. Positive effects of this antimicrobial combination against *Salmonella *points towards possible *in-vivo *use of the probiotic *L. plantarum *with co-trimoxazole for effective treatment of *Salmonella *infections

## Competing interests

The authors declare that they have no competing interests.

## Authors' contributions

PR conceived, supervised the study and finalized the manuscript. SP and PK performed the experiments and contributed to the writing of the manuscript. All authors read and approved the final manuscript.
